# A national-scale database of groundwater level data for Switzerland

**DOI:** 10.1038/s41597-026-07353-6

**Published:** 2026-05-14

**Authors:** Raoul A. Collenteur, Joaquin Jimenez-Martinez, Mario Schirmer, Christian Moeck

**Affiliations:** 1 Department of Water Resources and Drinking Water, Eawag, Swiss Federal Institute of Aquatic Science and Technology, 8600 Dübendorf, Switzerland; 2 Department of Civil, Environmental and Geomatic Engineering, ETH Zürich, 8093 Zürich, Switzerland; 3 Swiss Groundwater Network (CH-GNet), Dübendorf, Switzerland; 4 Present Address: Collenteur Hydroconsult GmbH, 3700 Spiez, Switzerland

**Keywords:** Hydrology, Hydrology

## Abstract

Groundwater is a vital component of the global supply of freshwater, playing a critical role for human populations, agriculture, and ecosystems. Due to the complex interactions between groundwater, surface water, climate, and human activity, these systems are frequently studied using advanced data analysis and modeling techniques. The effectiveness of these methods is generally enhanced by the availability and quality of data. In Switzerland, the focus area of this study, groundwater data is fragmented and lacks a standardized nationwide compilation. Consequently, the process of conducting nationwide studies with substantial sample sizes is both resource-intensive and time-consuming. In this paper we introduce the Swiss Groundwater Database, a comprehensive compilation of groundwater time series and associated metadata throughout Switzerland. The current database consists of groundwater level data from 985 monitoring wells, which were completed with additional static and time-varying variables. The environmental characteristics and climate indices were compiled and determined for each monitoring well. The database is designed to facilitate and support large-sample hydrological research related to groundwater in Switzerland and beyond.

## Background & Summary

Groundwater is a critical global source of drinking water, essential for agricultural irrigation and for sustaining ecosystems through its contribution to river and wetland baseflows^1^. To monitor and understand its temporal dynamics and overall status, measurements of groundwater levels (or hydraulic heads) serve as the most widely used source of subsurface information^2,3^. These measurements can be used to understand the complex relationships between climate, humans, subsurface, and groundwater dynamics^4,5^. This information can be used to make informed decisions about the sustainable management of groundwater resources. As big data analysis and artificial intelligence methods become increasingly prevalent^6^, large-sample groundwater hydrology is becoming a more common approach to effectively exploring interactions between groundwater, climate, ecosystems, and human societies.

The success of large-sample hydrological studies depends on access to extensive datasets that ideally span diverse regions, aquifer types, and climatic conditions. Moreover, the data should be homogenized to ease analyses. The surface water hydrology community has successfully taken up this grand challenge by developing large-scale databases, primarily focused on streamflow, and increasingly incorporating catchment-level metadata such as land use, soil properties, and climate variables. A good example of these efforts is the family of CAMELS (Catchment Attributes and Meteorological time series for Large Samples) datasets, which contain hydrometeorological time series along with catchment attributes and hydrological signatures^7,8^. Meanwhile, over a dozen CAMELS datasets have been developed for multiple countries and regions, also culminating in the CARAVAN global dataset^9^. The substantial number of citations suggests these datasets are being used, and support a broad range of scientific studies.

Large, homogenized databases of groundwater data are less common, but the groundwater community is quickly catching up. Moeck *et al*.^10^ created a global-scale database of groundwater recharge estimates, a vital flux of water into groundwater systems. Olarinoye *et al*.^11^ developed a global database of spring discharge hydrographs. In recent years, multiple studies have developed larger databases for groundwater levels. For example, Lin *et al*.^12^ created a database of groundwater level measurements in the US, Venegas- Quiñones *et al*.^13^ for Chile, Wang *et al*.^14^ for China, Uchôa *et al*.^15^ for Brazil, Chávez García Silva *et al*.^16^ for southeastern Europe (Portugal, Spain, France, and Italy), and Ohmer *et al*.^17^ presented a large-sample database of groundwater levels for Germany. A substantial effort to collect groundwater level data globally is led by the International Groundwater Resources Assessment Centre^18^. This dataset has been expanded into the GROW (global integrated GROundWater) dataset^19^, which includes other attributes and meteorological time series.

Although the latter two include a few dozen wells from Switzerland, the overall coverage for Switzerland and neighboring countries remains limited (±30 wells in Switzerland). This is likely because data is collected at different government levels and not centrally stored. Switzerland is of particular interest to hydro(geo)logists because it is a key headwater region supplying downstream areas in Europe^20^ and because it exhibits a large geological complexity and landscape diversity over a relatively small area^21^. Strong gradients in elevation, climate, and land use, together with heterogeneous hydrogeological settings (from alpine bedrock to productive Quaternary aquifers), create a wide range of recharge regimes and groundwater-surface water interactions. The presence of snow- and glacier-influenced hydrology and high anthropogenic pressure in populated valleys further increases the value of Switzerland as a natural laboratory for evaluating monitoring strategies and large-sample groundwater analyses.

In this paper, we present the Swiss Groundwater Database: a database of groundwater level and meteorological time series, environmental and climatic descriptors, and groundwater signatures for Switzerland. This database aims to support small- and especially large-sample groundwater hydrology research in Switzerland and beyond, by combining quality-controlled time series of groundwater levels and meteorological drivers into a single dataset. Importantly, groundwater level data collected and quality-controlled by the Federal Office for the Environment (FOEN) and the cantons (member states of the Swiss Confederation) were combined into a single resource. These data previously existed only in fragmented, non-centralized formats that were not all publicly available. Furthermore, a preprocessed (i.e., gap-filled and aligned with the meteorological drivers) dataset of groundwater level data is provided, suitable for analyses requiring time series with equidistant time intervals between the measurements. In addition to enabling scientific studies, the database aims to improve data accessibility and interoperability, support evidence-based groundwater management and policy, enable cross-regional and transboundary analyses, and provide a consistent basis for model calibration and validation.

The database comprises over 985 groundwater level time series from across Switzerland that have passed the minimum data requirements and the quality control process and are provided as the core dataset. For studies and analyses requiring gapless time series, imputed time series are provided for the period 1980-2023. The data was augmented by collecting meteorological time series (i.e., precipitation and air temperature) from the Federal Office of Meteorology and Climatology (MeteoSwiss), calculated climatic forcings (i.e., potential evaporation), snow information from the SPASS dataset^22^, as well as climatic (i.e., number of snow days) and environmental descriptors (i.e., soil texture) for each monitoring well. All data and scripts are available from the Zenodo repository as FAIR data: 10.5281/zenodo.14260400^23^. Future developments aim to 1) complement the current database with data from more groundwater monitoring wells (particularly in missing regions) and 2) enhance the database by incorporating additional groundwater-relevant parameters and information—such as spring discharge, groundwater quality data, or aquifer characteristics (i.e., porosity, hydraulic conductivity, and transmissivity)—to further improve interpretability and support more nuanced comparisons. The authors intend to update the database regularly with new data and welcome community additions to the Swiss Groundwater Database.

## Methods

### Groundwater level data

The 26 cantonal offices and the Federal Office for the Environment (FOEN) in Switzerland were contacted with a request to share groundwater level time series data for the database. The data requirements were kept to a minimum, with the practical goals of 1) minimizing the workload for the responsible offices, and 2) maximizing the amount of data available in the database. Specifically, the offices were asked to provide quality-controlled time series of groundwater levels, with (preferably daily) measurements between 1900 and 2023 for a minimum period of 5 years and 60 measurements (12 per year). All measurements are above mean sea level (amsl). No restrictions were made on the measurement frequency of the data, as this traditionally shows large variations over time (e.g., different measurement techniques) and space (e.g., different data collection frequencies between cantons). Importantly, the data from each monitoring well had to be accompanied by latitude and longitude values (or equivalent) to be able to geolocate each time series. Finally, data providers were requested to deliver validated records that had been reviewed for errors and outliers, establishing a consistent quality foundation before applying our own quality control procedures.

### Groundwater level data imputation

Data gaps and irregular time intervals between groundwater level measurements are common, arising from technical limitations, logistical constraints, or irregular sampling practices. These characteristics can hinder, for example, resource assessment, the development of process understanding, and predictive modeling^24^. Although various methods have been developed to cope with these common characteristics, for example, for model calibration^25^, trend analysis^26^, and when using input data with gaps in deep learning^27^, many analysis methods require groundwater level time series with equidistant time intervals between the measurements to be applicable. To increase the general usability of the dataset for such methods, a gap-filled version of the database is therefore also provided.

Gaps in groundwater level time series were imputed using the ARCHI (Automated Regional Correlation Analysis for Hydrologic Record Imputation) method, implemented in the ARCHI R package. ARCHI aggregates the groundwater level observations to a user-defined common timestep, builds correlation-based linear models between wells, and iteratively imputes missing values while enforcing explicit model-fitness criteria. The algorithmic details of ARCHI are described in Levy *et al*.^28^, and the workflow used is shared with the dataset. Gapless time series were constructed for the period 1980-01-01 to 2023-08-31.

### Meteorological data

For each monitoring well, meteorological data were collected and obtained from MeteoSwiss. Specifically, daily precipitation [mm d^−1^], daily mean, minimum, and maximum temperature data [°C], and daily relative sunshine duration are taken from the RhiresD, TabsD, TminD, TmaxD, and SrelD raster datasets, respectively. The gridded datasets are based on interpolated station data. For each groundwater monitoring station, the meteorological data is extracted from the grid cell in which the station is located. All meteorological data is available for the period from January 1971 to August 2023.

The potential evaporation [mm d^−1^] is subsequently computed using the Hamon equation^29^ as implemented in the Python package PyEt^30^ from the temperature data. The daily time series of the meteorological variables are also provided as part of the database. The provision of this data makes it easier to analyze and model groundwater level data in the context of these meteorological variables and climatic changes, as was, for example, done in Collenteur *et al*.^31^ for groundwater level data in Switzerland.

### Snow height and SWE data

Data about snow depth and snow water equivalent, presumably important factors influencing groundwater level fluctuations in alpine regions, are extracted from the gridded SPASS dataset^22^ provided by the Institute for Snow and Avalanche Research (SLF). The data is computed using a quantile-mapped temperature index model that was evaluated against station data, with generally good results^22^. Nonetheless, it is noted here that the provided values are simulated values and not station measurements. Again, time series for each groundwater station are extracted from the grid cell in which the station is located. Similar to the meteorological data, the data is available for the period from January 1971 to August 2023.

### Environmental and climatic descriptors

A range of environmental and climatic descriptors was derived for each monitoring well, such as well locations, mean meteorological data from 1971 to 2023 (precipitation and temperature), and additional relevant geospatial datasets (i.e., lithology). These descriptors provide information about the environmental setting of the different monitoring wells and can be used in future studies to explain observed groundwater behavior and enhance modeling efforts. Similar auxiliary information is provided in datasets such as CAMELS^7^, which facilitates the joint use and comparison of these datasets in large-sample hydrological analyses. An overview of the environmental and climatic descriptors is shown in Table 1, and the data sources for the individual descriptors are further discussed below. All descriptors are provided as static variables.

**Table 1:** Environmental and climatic descriptors that are computed for each groundwater monitoring well.

Descriptor	Abbreviation	Unit	Data Source
Latitude	lat	°	—
Longitude	lon	°	—
Altitude	alt	m a.s.l.	SwissALTI3D
Slope class	slope_class	—	SwissALTI3D
Start date	start_date		—
End date	end_date	—	—
Depth to water table	dtw	m	—
Distance to nearest river	dtr	m	SwissTLM3D
Mean annual precipitation	p_mean	mm	MeteoSwiss
Mean annual pot. evaporation	pet_mean	mm	MeteoSwiss
Mean annual temperature	t_mean	°C	MeteoSwiss
Number of snow days	snow_days	days	MeteoSwiss
Aridity Index	aridity	—	—
Lithology	lithology	—	swisstopo^32^
Tectonic unit	tectonic	—	swisstopo^32^
Genesis	rocktyp	—	swisstopo^32^
Soil texture	soil_texture	—	SoilGrids^34^
Land use	landuse	—	CORINE^35^
Groundwater occurrence	hydroavailability	—	FOEN^33^
Hydrogeology	aquifer_type	—	FOEN^33^

The altitude of the land surface at the monitoring well locations was extracted from the SwissAlti3D terrain model from the Swiss Federal Office of Topography swisstopo using the latitude-longitude data provided from the well location. SwissALTI3D is a highly precise digital elevation model that represents Switzerland’s terrain surface without vegetation or built structures, provided as a grid with a resolution of 0.5 or 2 m, and delivered in complete 1 km^2^ tiles. The slope classes were uniformly calculated. The slope data are based on the SwissAlti3D model from swisstopo. The following slope categories were defined:  <18%, 18–35%, 35–50%, and ≥50%. Slope areas less than 100 m^2^ were excluded. The depth to the water table is computed as the altitude of the land surface minus the average groundwater level (note the positive sign). The distance to the river is computed as the length of the path to the most nearby river in meters, using GIS data (SwissTLM3D) from the river network obtained from swisstopo. This metric provides a simple national-scale proxy for potential groundwater-surface water interaction but does not account for hydraulic connectivity, river stage, aquifer geometry, subsurface flow paths, or geological controls.

The lithological and geological data of Switzerland are provided by swisstopo^32^. Data include the distribution and tectonic classification of the uppermost sediment and rock layers, major structural units, and groundwater resources with their yields and occurrence. The Groundwater Occurrence map^33^ classifies near-surface groundwater resources based on permeability, which depends on lithology and structural characteristics and is divided between groundwater resources in consolidated and unconsolidated rocks. The soil textures were obtained from the SoilGrids dataset at a 250 m resolution^34^.

Land use was estimated using the CORINE Land Cover (CLC) datasets^35^ from the European Copernicus program. Geo-referenced data with a resolution up to 100 m were used to extract land cover types with the most recent dataset from 2018.

### Groundwater signatures

To characterize the behavior captured in each groundwater level time series, a set of groundwater signatures is computed for every record in the dataset. Groundwater signatures, also known as indices, are quantitative scalar metrics that capture and describe key characteristics of groundwater level time series^36,37^. Examples of groundwater signatures include the average rise rate, which reflects the typical speed of groundwater level increases; the autocorrelation time, indicating the temporal persistence or memory of the system; the Pardé seasonality index, which quantifies the regularity and strength of seasonal fluctuations; and the interannual variation, measuring variability in groundwater levels from year to year. In total, 27 groundwater signatures are computed for each groundwater level time series, using the weekly means of the data from 1990 to 2020. Explanations and further references for individual signatures can be found in Collenteur *et al*.^37^ and on the Pastas documentation website (www.pastas.readthedocs.io). Signatures can, for example, be used to analyze data, compare and select time series, cluster monitoring wells, and improve machine and deep learning models to model groundwater levels by using them as static features.

## Data Records

The data described in this manuscript can be accessed at Zenodo^23^: 10.5281/zenodo.14260400. The repository^23^ also includes the scripts to process the data and reproduce the figures for this manuscript. An example Python script of how to load the data into convenient Pandas DataFrames is provided as well. An overview of the files in the dataset is provided in Table 2.

**Table 2:** Overview of the provided data files and contents.

File/Folder name	Description
heads.csv	The time series of groundwater level data, with the date as the index and the ID as the column name. The dates on which no measurements were recorded are left empty.
imputed	Folder with the gap-filled time series for each canton separately, available from 1980 to 2023.
precipitation.csv	The precipitation time series for each well with the dates as indices and one column for each monitoring well.
temperature.csv	The air temperature time series for each well with the dates as indices and one column for each monitoring well.
evaporation.csv	The potential evaporation time series for each well, with the dates as indices and one column for each monitoring well.
descriptors.csv	The environmental and climatic descriptors for each monitoring well, including metadata information of the monitoring wells with latitude, longitude, and altitude.
signatures.csv	The groundwater signatures for each monitoring well.
snow_height.csv	The daily snow height in meters for each monitoring well.
swe.csv	The snow water equivalent in meters for each monitoring well.

All data are provided in human-readable comma-separated variable (CSV) files for easy and fast import and data processing. Each monitoring well is assigned a unique identification (ID) code, based on the original code used by the data provider and an abbreviation of the data provider. This ID is used throughout all the files to identify the monitoring well. The tables are all provided in wide format, with IDs as columns and variables per row. A detailed description of the dataset is presented in the following sections.

## Data Overview

### Groundwater data

**Fig. 1:**
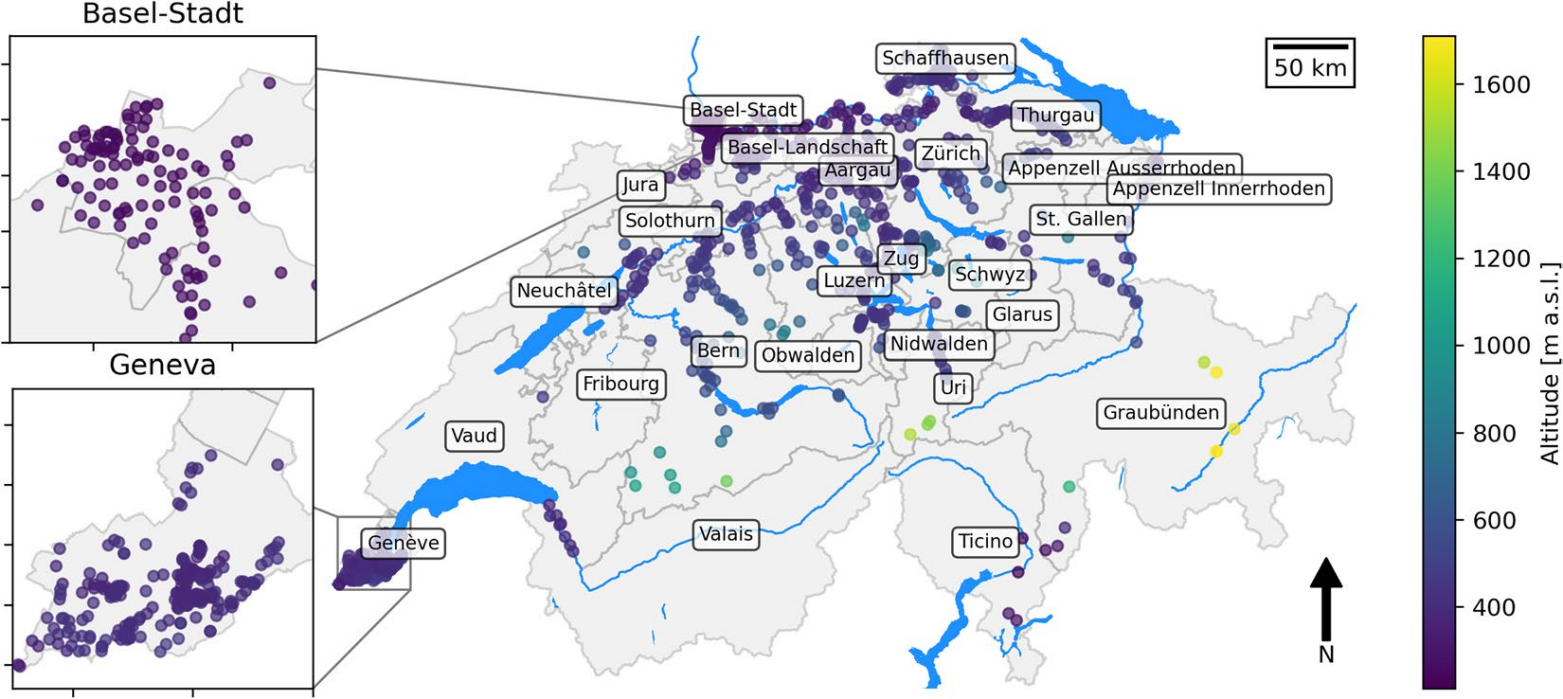
Locations of the monitoring wells in the database, colored by the altitude of the land surface at the monitoring well. The labels denote the different cantons of Switzerland.

Figure 1 shows a map with the locations of the monitoring wells included in the dataset after quality control. The color of the data points denotes the altitude of the land surface at the location of the monitoring well. The dataset covers a substantial portion of Switzerland, with most of the monitoring wells located in the northern regions. This area, also known as the Swiss Plateau, is the most densely populated region of the country and is characterized by intensive agricultural activity. In contrast to the Alpine regions, it features extensive alluvial aquifers that are more suitable for the installation of piezometers and represent groundwater resources of greater practical relevance for regional water use. The combination of favorable hydrogeological conditions and the increased demand for groundwater monitoring—driven by higher population density and land use pressure—likely explains the denser distribution of wells in these areas. The areas where the effect of urbanization on the density of groundwater monitoring networks is particularly clear are, for example, the canton of Geneva and Basel-Stadt, as shown in the inset plots in Fig. 1.

The spatial distribution of the monitoring wells can be linked to the elevation of the land surface, with fewer wells at increasing altitudes in the Alpine region. This is in part the result of the hydrogeological settings of the area, which is dominated by hard and fractured rock aquifers, where it is substantially more difficult to install monitoring wells and groundwater resources are typically more localized and discontinuous. Moreover, water supply in these areas is more dependent on spring discharge, which is not (yet) part of this database. It is worth noting, however, that many monitoring wells are located in alluvial aquifers of mountain valleys (i.e., the Rhone, Inn, and Rhine valleys) in the south and east. Another important reason for the spatial data gaps is that no data was provided or that the time series were too short for some regions. For some of these regions without data, the FOEN data provides some coverage. Already, the database covers a substantial portion of Switzerland, and future updates are expected to further expand its spatial coverage, helping to close remaining gaps and achieve even broader national representation. However, a completely uniform spatial distribution of monitoring wells is unlikely, reflecting the uneven distribution of groundwater resources and Switzerland’s geological and topographic complexity.

#### Temporal availability

**Fig. 2:**
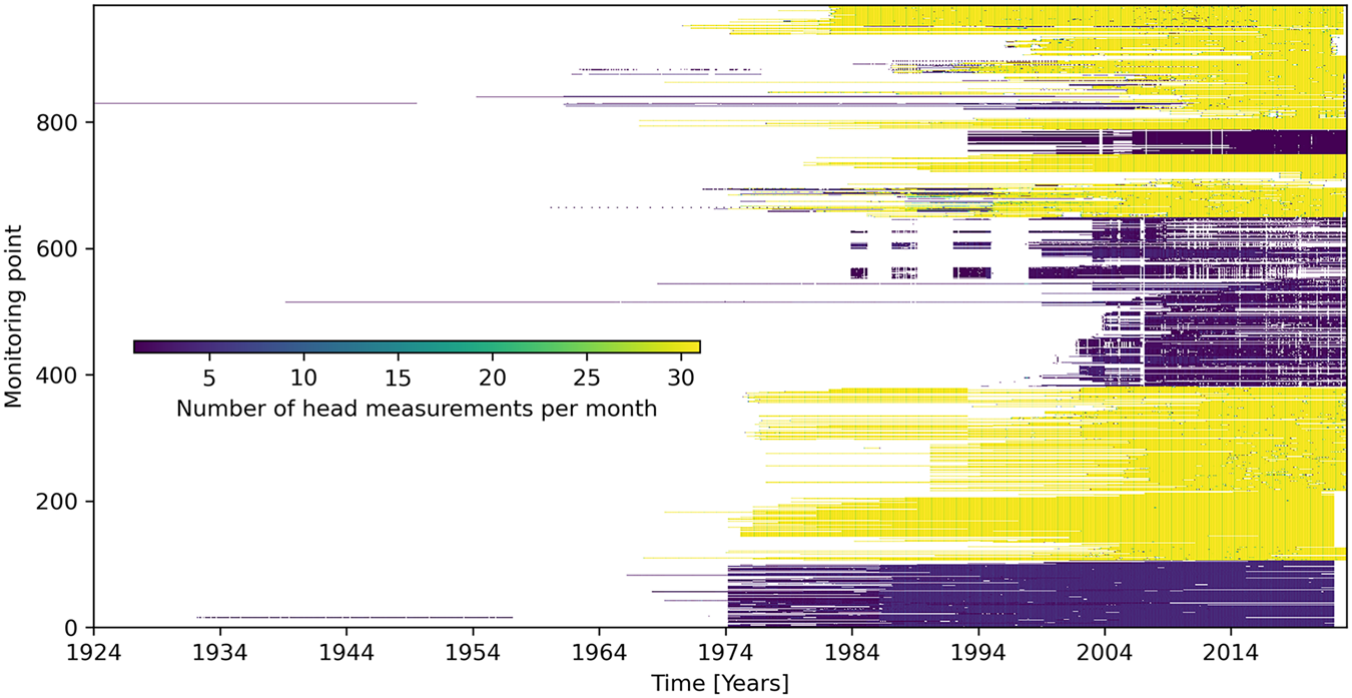
Data availability of the groundwater level time series. Each row is a time series, and the color denotes the temporal resolution of the data.

Figure 2 shows the availability of data for all 985 well locations. Each row is a time series, and the color scale indicates the number of measurements per month, where yellow denotes months with a high number of observations (e.g., daily measurements), while purple indicates months with only a small number of measurements. The first measurement in the database was made on the 15th of November in 1924, and the number of wells gradually increased over time. In particular, from the 1970s onward, the number of available measurements increased sharply, possibly related to the Federal Water Protection Act [Gewässerschutzgesetz, GSchG^38^], which may have led to increased monitoring efforts. The majority of the dataset thus focuses on more recent decades, and only a few time series provide longer-term information. The measurement period ranges between 5 (the minimum length requirement) and 100 years, with an average length of 28 years of data. Figure 2 shows that the measurement length is highly variable, and there are relatively few measurements before 1970.

Throughout the measurement period, the method to measure groundwater level may have changed for individual monitoring wells (i.e., purple goes to yellow). For example, the measurements were first conducted manually and later with a pressure transducer^3^. As a result, the temporal resolutions of the groundwater level measurements change over time for many monitoring wells. The most common measurement frequencies are once per day, once per week, and once per month. As is common in groundwater data, many of the groundwater level time series have irregular time steps between the groundwater level measurements. These data collection results also highlight the need to deal with irregular time steps between measurements when analyzing and modeling (long-term) groundwater data. Based on this characteristic, different subsets of the data may have to be gathered from this data set for formal analyses, that is, if analysis methods require constant time steps between measurements.

A gap-filled version of the data from 1980 to 2023 is provided as well for analyses requiring equidistant time steps. Because the original data have varying frequencies between the cantons (i.e., daily, weekly, or monthly data), the gap-filling algorithm is run for each canton separately. Consequently, the imputed data is provided for each canton separately with the highest possible frequency. It is stressed here that many methods of data imputation exist, and the results of subsequent analyses using the gap-filled data will depend on the method chosen. The imputation performance metrics for the gap-filling models generally show good agreement with the observed data across cantons (i.e., the median NSE over all wells was above 0.75). Nonetheless, we strongly suggest that, where possible, for example, for tasks such as model evaluation and process-based analyses, the original measurements rather than the gap-filled data are used.

### Environmental and climatic descriptors

**Fig. 3:**
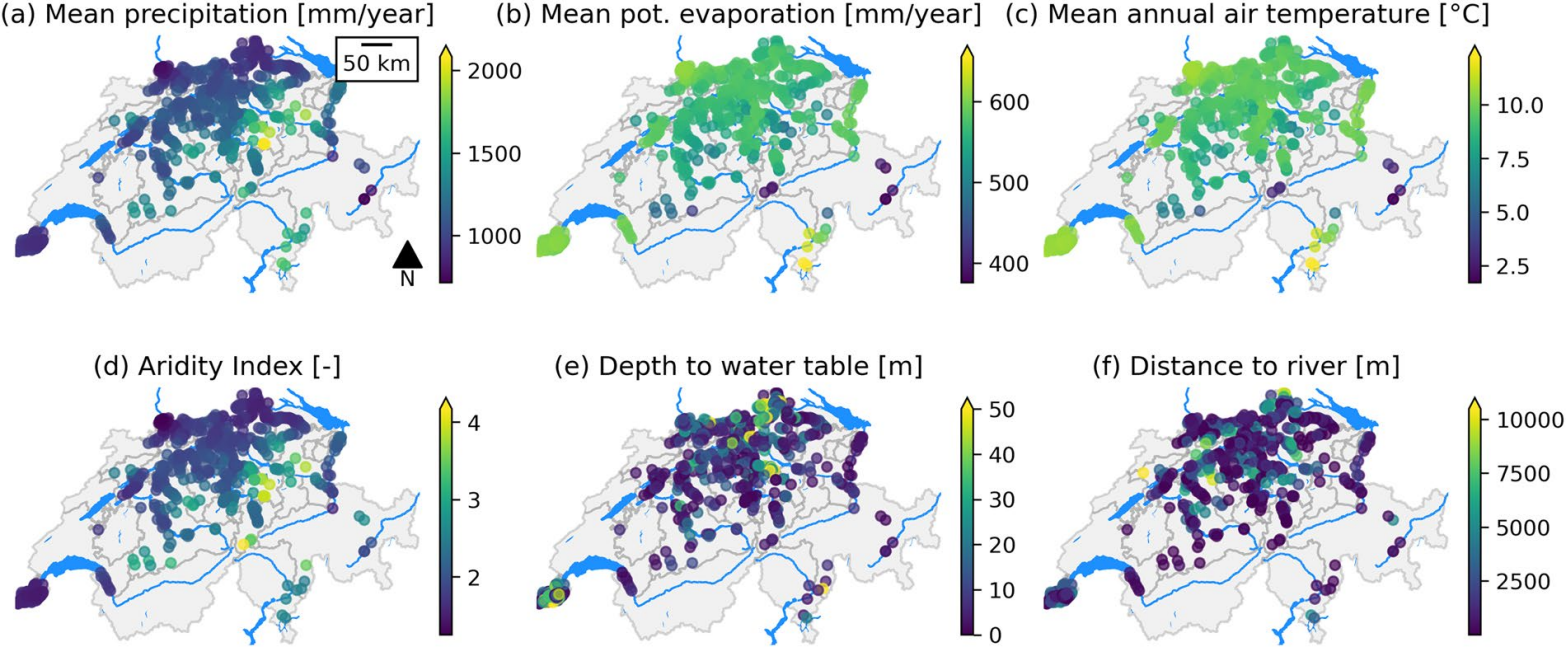
Maps with examples of various environmental and climatic descriptors for the study area.

Figure 3 shows overview maps for six of the environmental and climatic descriptors that were collected. For the climate descriptors, Fig. 3 shows the average temperature (Fig. 3a), the annual precipitation sum (Fig. 3b), the annual potential evaporation (Fig. 3c), and the Aridity Index (Fig. 3d), all calculated for the 30-year period 1993-2023. The data shown in these figures indicates that there is some variability in the data, particularly from north to south. The largest amount of precipitation is measured in the Alps, which also coincides with lower temperatures and potential evaporation. Based on the Aridity Index (AI), all of Switzerland is classified as humid. Nonetheless, spatial variability is evident, with higher AI values in inner-Alpine valleys and southern regions and lower values across the Swiss Plateau and northern Alpine foreland, reflecting strong topographic and climatic gradients.

For environmental descriptors, Fig. 3 shows the depth to the water table (DTW, Fig. 3e) and the distance to the river (Fig. 3f). For the largest portion of the wells, the depth to the water table is relatively small, on the order of meters up to twenty meters. Only a small percentage (~17%) has a depth to the water table exceeding 20 meters. Similarly, most monitoring wells are located within a few kilometers of a river—an expected pattern, as many are situated in unconsolidated alluvial aquifers that typically develop along river corridors and are hydraulically connected to surface water systems.

### Groundwater dynamics

**Fig. 4:**
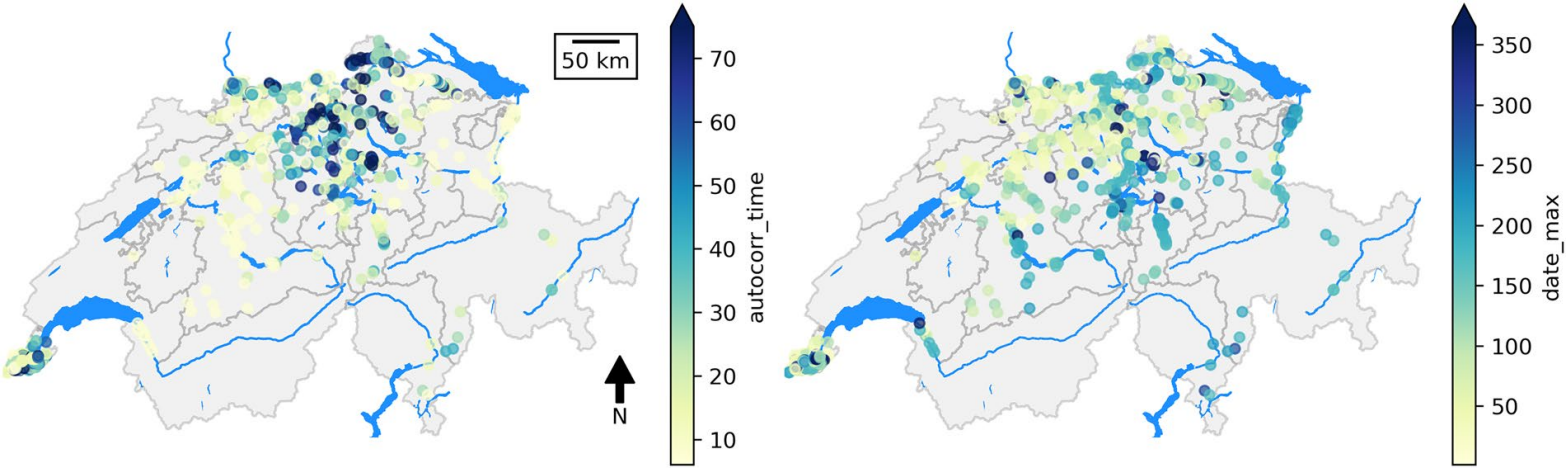
Examples of two of the groundwater signatures for the monitoring wells. The left plot shows the autocorrelation time, and the right plot shows the average day of the year with the maximum groundwater level.

For each of the 985 groundwater level time series, 27 groundwater signatures were computed to characterize groundwater behavior at each monitoring well. Figure 4 shows examples of the values of two groundwater signatures. The plot on the left shows the autocorrelation time, the number of days it takes the autocorrelation function to drop below 0.9. This is interpreted as a measure of the memory of the system [e.g.^39^]. The larger the value (i.e., number of days), the larger the memory. Higher values (blue) indicate slower, more persistent groundwater responses, while lower values (yellow) reflect more dynamic systems. A spatial pattern emerges, with longer autocorrelation times predominantly found in the northern lowland regions, particularly the Swiss Plateau, where larger unconsolidated aquifers are common. In contrast, shorter autocorrelation times are more frequent in alpine and southern areas, likely reflecting more dynamic groundwater systems with faster responses to recharge events.

The plot on the right shows the average day of the year with the maximum groundwater level having a clear spatial pattern. Particularly in the northeastern part of Switzerland, the groundwater level peaks early in the year (i.e., January to March), while towards the Alps, the groundwater level peaks later (April-June). These patterns can be partially linked to the dominant recharge processes, where winter precipitation in the Alps falls more as snow and melts in spring, while in the north, the winter precipitation infiltrates and causes an earlier rise and subsequent peaking^40^. Another probable reason is the influence of the rivers that also peak in spring. Future research can further investigate the relationships between groundwater behavior and descriptors, such as those shown above.

## Technical Validation

**Fig. 5:**
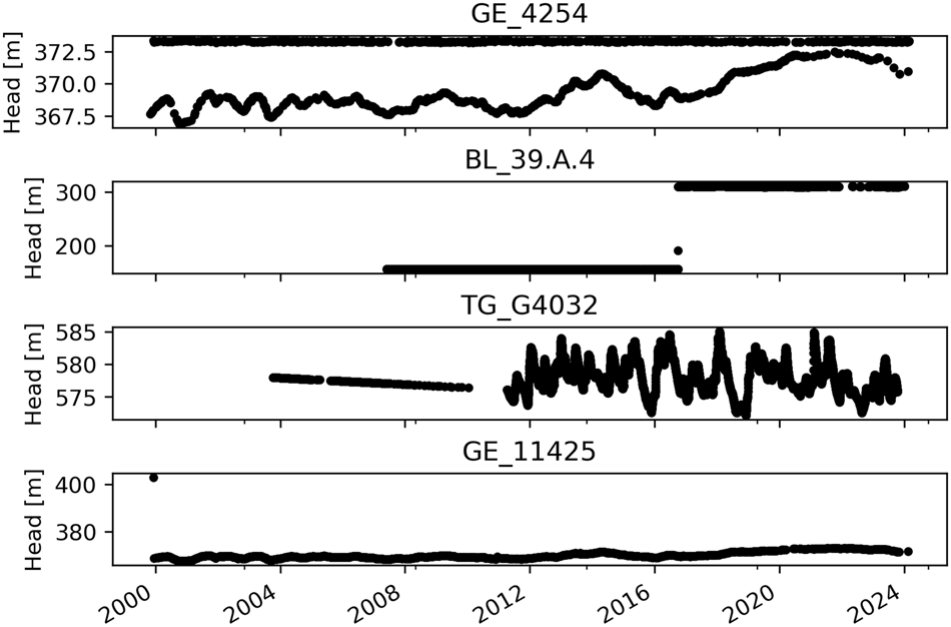
Examples of groundwater level time series that were removed from the database (**a** and **b**), or where part of the data was removed (**c** and **d**).

In total, 19 out of 26 cantonal offices and the FOEN provided data for the database (1185 time series in total). The data from 1000 monitoring wells (i.e., 1000 time series) met the initial criteria. Only quality-controlled data were requested from the providers, but a preliminary screening revealed that some inconsistencies and errors remained in a few of the time series. In addition to the quality control from the data providers, each individual time series was therefore visually checked to remove clear outliers and obvious data errors. Only time series with obvious errors were (partly) removed. Figure 5 shows four examples of groundwater level time series that were removed (Fig. 5a,b) or where part of the data was identified as an outlier and removed (Fig. 5c,d). The first time series shows unusual measurement patterns that cannot be readily explained. The presence of extended segments with constant values suggests potential data artifacts. The second time series shows a big, unexpected jump in the data. The third time series shows an example of interpolated data at the beginning of the time series. The fourth time series shows a clear outlier at the beginning of the time series. In total, 32 time series showed unexpected values. Of these, 15 were removed from the dataset because there are no viable options to correct the data (i.e., as in Fig. 5a,b). For the other 17 time series, the outliers, or time periods, with interpolated data were removed (i.e., as in Fig. 5c,d). The database currently contains 985 time series, a number that is intended to grow as more data is added over time.

## Data Availability

The dataset is available from the following Zenodo repository^23^: 10.5281/zenodo.14260400.

## Code availability

All the code to preprocess the data and reproduce the figures in this paper is available from the Zenodo repository^23^: 10.5281/zenodo.14260400.
